# *Urtica dioica* Leaf Infusion Enhances the Sensitivity of Triple-Negative Breast Cancer Cells to Cisplatin Treatment

**DOI:** 10.3390/ph16060780

**Published:** 2023-05-23

**Authors:** Guy Nafeh, Maria Abi Akl, Jad Samarani, Rawane Bahous, Georges Al Kari, Maria Younes, Rita Sarkis, Sandra Rizk

**Affiliations:** 1 Department of Natural Sciences, Lebanese American University, Byblos P.O. Box 36, Lebanon; guy.nafeh@lau.edu (G.N.); maria.abiakl01@lau.edu (M.A.A.); jad.samarani@lau.edu (J.S.); rawane.bahous@lau.edu (R.B.); georges.alkari@lau.edu (G.A.K.); maria.younes01@lau.edu (M.Y.); rita.sarkis@epfl.ch (R.S.); 2 Laboratory of Regenerative Hematopoiesis, Swiss Institute for Experimental Cancer Research (ISREC) & Institute of Bioengineering (IBI), School of Life Sciences, Ecole Polytechnique Fédérale de Lausanne (EPFL), 1015 Lausanne, Switzerland

**Keywords:** *Urtica dioica*, breast cancer, apoptosis, cisplatin, combination therapy

## Abstract

*Urtica dioica* (UD) has been widely used in traditional medicine due to its therapeutic benefits, including its anticancer effects. Natural compounds have a promising potential when used in combination with chemotherapeutic drugs. The present study explores the anticancer and anti-proliferative properties of UD tea in combination with cisplatin on MDA-MB-231 breast cancer cells in vitro. To elucidate the effect of this combination, a cell viability assay, Annexin V/PI dual staining, cell death ELISA, and Western blots were performed. The results showed that the combination of UD and cisplatin significantly decreased the proliferation of MDA-MB-231 cells in a dose- and time-dependent manner compared to each treatment alone. This was accompanied by an increase in two major hallmarks of apoptosis, the flipping of phosphatidylserine to the outer membrane leaflet and DNA fragmentation, as revealed by Annexin V/PI staining and cell death ELISA, respectively. DNA damage was also validated by the upregulation of the cleaved PARP protein as revealed by Western blot analysis. Finally, the increase in the Bax/Bcl-2 ratio further supported the apoptotic mechanism of death induced by this combination. Thus, a leaf infusion of *Urtica dioica* enhanced the sensitivity of an aggressive breast cancer cell line to cisplatin via the activation of apoptosis.

## 1. Introduction

Breast cancer has replaced lung cancer as the most diagnosed cancer worldwide with a total of 2.3 million new cases in 2020 [1]. It is also the most common kind of cancer in the Middle East, with 2.26 million cases reported in the year 2020 [2]. More specifically, Lebanon has the sixth highest age-standardized incident rate for breast cancer in the world [3]. Due to changes in risk factor profiles, improved cancer registration, and cancer detection, its incidence and death rates have risen over the past three decades [4]. These numbers are projected to increase and reach 3 million cases annually by the year 2040 [5]. Breast cancer can be characterized by molecular subtypes according to the receptors found on their surface. The triple negative MDA-MB-231, used to model late-stage breast cancer, lacks the progesterone, estrogen, and HER2 receptors, making it one of the most aggressive types of malignancies [6,7]. Despite the better efficacy and increased survival afforded by modern treatments, the side effects and long-term repercussions of anti-cancer chemotherapy continue to be a significant cause of concern for both patients and healthcare professionals [8]. In fact, cancer patients may experience adverse effects from chemotherapy that are recurrent, potentially fatal, and frequently occur at home [9]. The most frequent side effects of chemotherapy are nausea and vomiting, exhaustion, decreased appetite, alterations in taste, loss of hair, dry mouth, and constipation [10]. It is therefore crucial to develop effective strategies for the management of the adverse side effects linked to chemotherapy. Hence, natural-based therapies have been a source of great interest in recent studies.

Stinging nettle, or *Urtica dioica* L., is an herbaceous perennial flowering plant that is native to Eurasia, a member of the Urticaceae family, and is used interchangeably in medicine [11]. This species is characterized by its sting and hairy, serrated leaves, making it easily identifiable from other species in the *Urtica* genus [12,13,14]. *Urtica dioica* (UD) leaves are also an excellent source of nutrients such as vitamins, minerals, polyphenols, amino acids, and fibers [15,16,17]. As such, it has been used in folk medicine to treat skin conditions including jaundice, burns and rashes [18,19], as well as internal bleeding, diabetes, kidney stones, anemia, and hypersensitivity [20]. More interestingly, stinging nettle was previously reported to be one of the most widely used herbs in complementary and alternative medicine for the treatment of cancer patients [21,22]. The traditional use of *Urtica dioica* in cancer chemoprevention dates to the 8th century as reported by Abu Darwish et al. [23]. In fact, several studies reported the usage of the *U. dioica* seeds and leaves as the most prominent plant parts for cancer treatment [24,25].

Stinging nettle young leaves have also long been utilized as a source of nutritive food, and different components are also employed as conventional remedies. It has a variety of bioactive phytoconstituents, including polyphenols, and is high in phytonutrients [26,27]. Strong antioxidative, anti-inflammatory, anti-ulcer, anti-hyperglycemic, anti-bacterial, and cardiovascular protective pharmacological actions have been demonstrated by UD preparations [28]. Additionally, recent studies have shown that the leaves of UD are an intriguing source of biologically active chemicals that can be used for human prophylaxis and therapy, supporting its historic use in the treatment of infectious disorders, diabetes, and arthritis [29].

Despite all of these investigated properties of UD extracts, proof of its anti-cancer activity is rare and restricted to a few in vitro studies on breast, prostate, and, most recently, lung cancer and leukemia cell lines, as well as few in vivo experiments that used a wide range of organic compounds of a UD leaf extract [30,31]. Our laboratory has a historical interest in the anticancer properties of edible UD extracts. In 2020, Hodroj et al. showed that the treatment of Acute Myeloid Leukemia (AML) cells with UD in vitro had a dose- and time-dependent antiproliferative effect. The cell-death ELISA and cell-cycle analysis assays showed that the extract activated apoptosis in the U937 cells via the Bax/Bcl-2 pathway and caused discontinuous DNA fragmentation [27]. 

Combination therapy, a chemopreventive strategy, has recently gained a new dimension with the potential use of natural compounds to enhance and support the activity of chemotherapeutic drugs [32]. Chemo-herbal drug combination therapy has been shown to increase therapeutic efficacy in preclinical studies by a variety of mechanisms, including induced apoptosis, decreased cell proliferation, cell cycle arrest, and interfering with important gene expression and protein signaling pathways in a variety of cancer cell lines [33]. Therefore, incorporating natural remedies into cancer treatment might be promising in reducing the previously mentioned adverse effects of chemotherapy [34]. In fact, the combinatory effect of the UD extract with chemotherapeutic drugs has been an area of great interest. *Urtica dioica* extract was shown to substantially increase the sensitivity of breast cancer cells to paclitaxel by decreasing the Snail-1 and the related gene expression [35]. Another study showed that UD also increased the sensitivity of lung cancer cells to cisplatin via endoplasmic reticulum-stress mediated apoptosis [36].

To our knowledge, no previous studies have assessed the combination effect of cisplatin and UD extract on breast cancer cells. Therefore, the aim of the current study is to investigate the combination effect of an infusion of *Urtica dioica* leaves, along with the chemotherapeutic drug cisplatin, on the triple negative breast cancer cell line MDA-MB-231. The goal is to use natural chemicals to lessen the overall toxicity of cisplatin in patients while promoting the anti-cancer efficacy of milder dosages of cisplatin.

## 2. Results

### 2.1. Antiproliferative Effect of Cisplatin and UD on Cell Proliferation 

**Figure 1:**
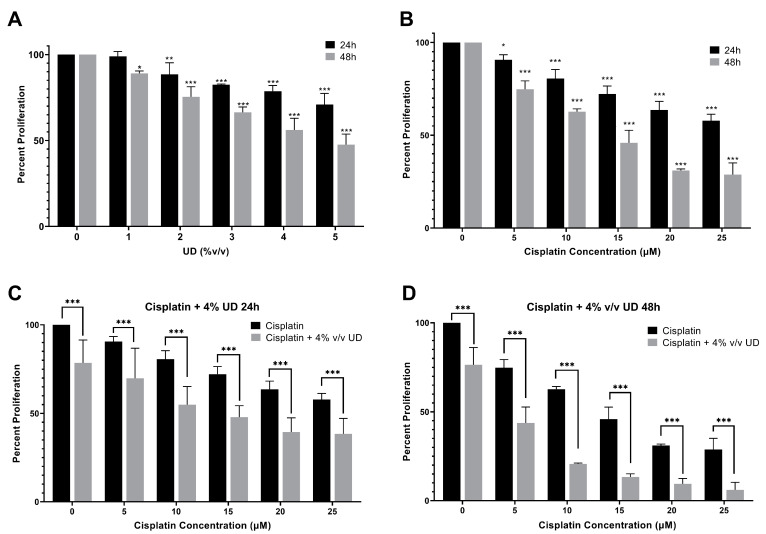
MTS cell viability assay. (**A**) Percent proliferation of the MBA-MB-231 cells after 24 and 48 h of treatment with different concentrations of UD (0–5% *v*/*v*). Results are compared to those of the untreated cells. Significant growth inhibition was observed on treatment with UD (*p* < 0.001). The IC_50_ was found to be 6.459% *v/v* at 24 h and 4.161% *v/v* at 48 h. (**B**) Percent proliferation of cells after 24 and 48 h of treatment with different concentrations of cisplatin (5–25 μM). A decrease in the proliferation of MDA-MB-231 cells was observed with increasing cisplatin concentrations. The IC_50_ was found to be 32.21 μM at 24 h and 12.76 μM at 48 h (*p* < 0.001). (**C**) Percent proliferation of cells upon treatment with increasing cisplatin concentrations in combination with 4% *v/v* UD after 24 h, (**D**) and 48 h. Results are compared to those of the cells treated with cisplatin alone. Each value represents the mean ± SD from three separate experiments; statistical analysis was determined by using Student’s t-test with * *p* < 0.5, ** *p* < 0.05, *** *p* < 0.001.

To quantify the effect of UD and cisplatin individually on cell proliferation, the MTS cell viability assay was performed. For that purpose, the MDA-MB-231 cells were cultured and treated first with gradient concentrations of cisplatin and UD extract for 24 and 48 h. Cisplatin and UD exhibited a dose- and time-dependent anti-proliferative effect when applied separately on the MDA-MB-231 cells. The results showed that UD significantly inhibited the proliferation of MDA-MB-231 (*p* < 0.001) in a dose- and time-dependent manner (Figure 1A), with an IC_50_ of 6.459% *v/v* at 24 h and 4.161% *v/v* at 48 h. As expected, a significant growth inhibition was observed upon cisplatin treatment (*p* < 0.001) (Figure 1B). The IC_50_ of cisplatin was determined to be 32.21 μM at 24 h and 12.76 μM at 48 h. Having shown that each of the UD and cisplatin exhibit antiproliferative effects on the MDA-MB-231 cells, we then aimed at evaluating their combination, as well as whether the UD extract can enhance the sensitivity of the TNBC cells to cisplatin treatment. Based on these results, subsequent experiments were performed using varying concentrations of cisplatin (5–25 μM) with 4% *v/v* UD, a concentration chosen to be less than the IC_50_ at 24 and 48 h. As reported above, UD (4% *v*/*v*) inhibited the proliferation of the MDA-MB-231 cells by approximately 20% after 24 h and 50–60% after 48 h, making it suitable for use in the combination treatment. 

### 2.2. UD Enhances the Sensitivity of the MDA-MB-231 Cells to Cisplatin

To determine the effect of the combination of UD and cisplatin on cell viability, increasing concentrations of cisplatin were combined with one concentration of UD at 4% *v*/*v*, as mentioned above. A significantly higher MDA-MB-231 inhibition was seen in the combination of UD and cisplatin at all concentrations when compared to cisplatin alone. UD in combination with 10 μM cisplatin at 24 h inhibited nearly half of the MDA-MB-231 cell proliferation when compared with 10 μM cisplatin alone (Figure 1C). The results also showed that the combination treatment significantly inhibited the proliferation of the MDA-MB-231 cells at all cisplatin concentrations used, with an IC_50_ of cisplatin being 13.17 μM in combination compared to 32.21 μM when used alone at 24 h (Figure 1C), and an IC_50_ of 4.207 μM in combination compared to 12.76 μM alone at 48 h (Figure 1D). Moreover, IC_50_ concentrations were further assessed by cytotoxicity assay, confirming the inhibition of 50% of cell growth as reported in the Supplementary Information (Table S1). The enhanced effect of UD (4% *v*/*v*) combination with cisplatin was revealed by the calculation of the combinatorial index (CI), which revealed an additive effect when combining cisplatin (15 and 20 μM) with 4% UD. Therefore, it can be inferred that UD significantly potentiated the inhibitory effect of cisplatin in breast cancer cells when combined with increasing concentrations of cisplatin [37].

### 2.3. The Combination of UD and Cisplatin Promotes Apoptosis in the MDA-MB-231 Cells

**Figure 2:**
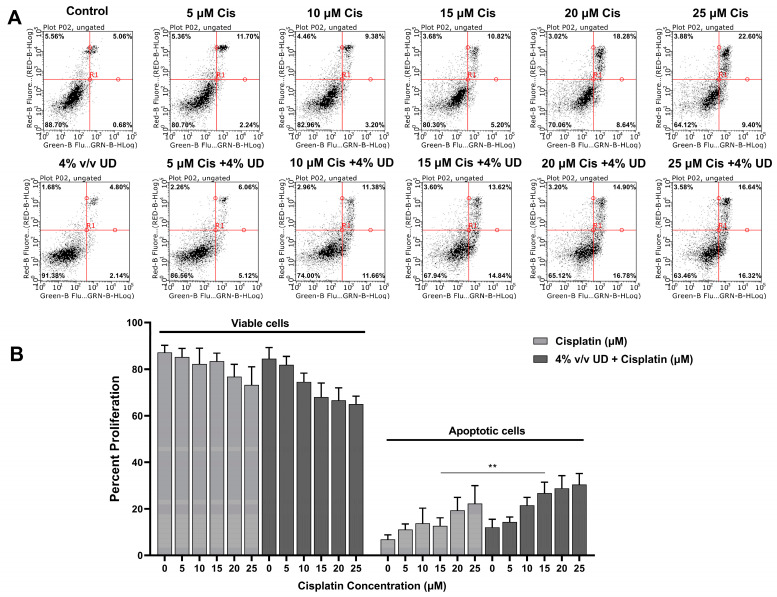
Dual Annexin V/PI staining of MDA-MB-231 cells. (**A**) Figures of the quantitative assessment of apoptosis on cells treated with 4% *v/v* UD and cisplatin (0, 5, 10, 15, 20, 25 μM) separately and in combination, stained with Annexin V/PI, and then analyzed using flow cytometry. (**B**) Histogram representing the means from 4 independent experiments +/− SD with **: *p* < 0.05.

To determine whether this combination enhances the activation of the apoptotic programmed cell death mechanism, Annexin V and PI were added to the triple negative breast cancer (TNBC) cells after treatment with both compounds separately and in combination. The cells were analyzed using a flow cytometer, and they were grouped into viable (Annexin^−^ and PI^−^) and apoptotic cells (Annexin^+^ and PI^+/−^) that appear in the lower left quadrant and in the two right quadrants, respectively (Figure 2A). As observed in Figure 2, increasing concentrations of cisplatin alone and cisplatin combined with 4% (*v*/*v*) UD both resulted in an increase in apoptotic cells. Interestingly, the most pronounced effect of UD in promoting the pro-apoptotic effect of cisplatin was detected upon exposure of MDA-MB-231 cells to 15 μM cisplatin, whereby the number of apoptotic cells was significantly increased from 12.685% when treated with cisplatin alone to 26.71% upon treatment with 4% *v/v* UD in combination with 15 μM cisplatin (Figure 2B). These results reveal the promising effect of the UD extract in enhancing the sensitivity of the TNBC cells to cisplatin treatment by further promoting the activation of apoptosis.

### 2.4. The Combination of UD and Cisplatin Increases DNA Fragmentation in the MDA-MB-231 Cells 

**Figure 3:**
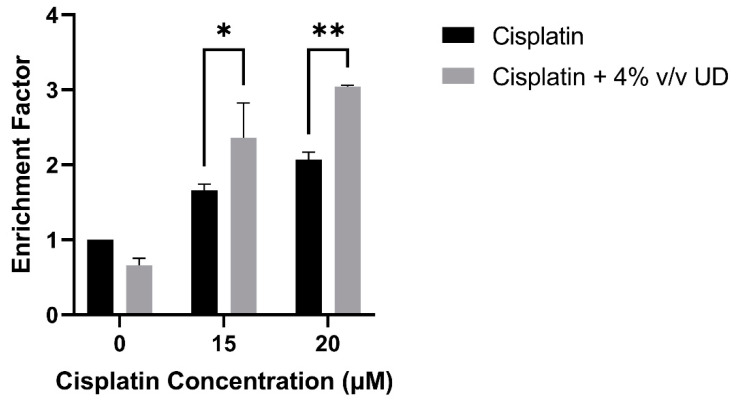
Cell-death ELISA showing the effect of 4% *v/v* UD extract on DNA fragmentation in MDA-MB-231 treated with 15 μM and 20 μM after 48 h. The graph represents the means from three independent experiments +/− SD. * *p* < 0.5, ** *p* < 0.05, respectively.

To quantify DNA fragmentation in the MDA-MB-231 cells treated with the combination of 4% *v/v* UD and cisplatin (15 and 20 μM), a cell death ELISA was performed. A significant dose-dependent increase in the enrichment factor, which is the ratio of absorbance compared to the control, was observed with the combination treatment compared to cisplatin alone. An approximate twofold and threefold increase was observed in the enrichment factor with the combination treatment compared to 15 μM and 20 μM cisplatin alone, respectively (Figure 3). These results further demonstrate that DNA fragmentation, a major hallmark of apoptosis, was significantly increased in the MDA-MB-231 cells by the combination of UD and cisplatin treatment.

### 2.5. The Combination of UD and Cisplatin Upregulates the Expression of Apoptotic Proteins

**Figure 4:**
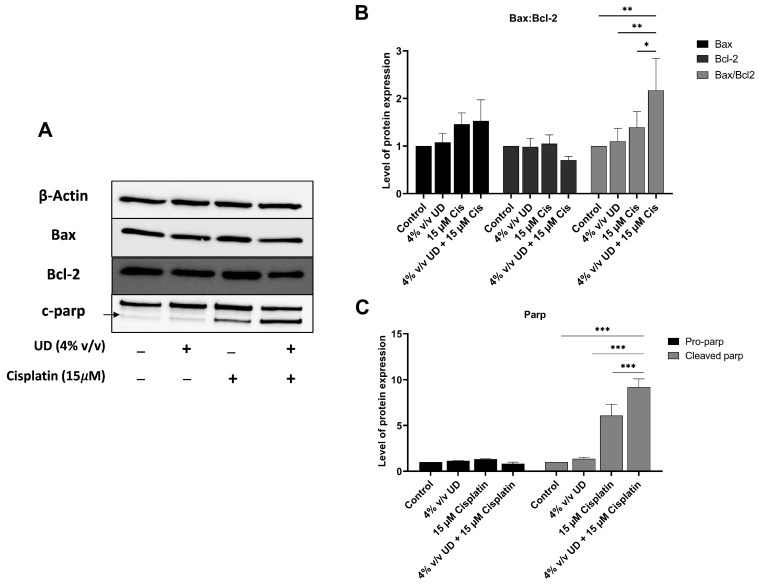
UD in combination with cisplatin activates the mitochondrial apoptotic pathway. (**A**) Western blots of apoptotic proteins. (**B**) Western blot analysis of apoptosis-regulating protein, Bax and Bcl2, in the MDA-MB-231 cells treated with concentrations 4% *v/v* UD, 15 μM cisplatin, and 4% *v/v* UD in combination with 15 μM cisplatin after 48 h. Significant upregulation of the Bax–Bcl2 ratio was observed with * *p* < 0.5, ** *p* < 0.05. (**C**) Western blot analysis of DNA-repair protein, PARP, in the MDA-MB-231 cells treated with 4% *v/v* cisplatin, 15 μM cisplatin, and combination of 4% *v/v* UD and 15 μM cisplatin. Upregulation of cleaved pro-apoptotic protein (c-PARP) expression was observed. Significant differences are reported, with *** *p* < 0.001.

To evaluate the combined effects of UD and cisplatin on the apoptotic pathway at a molecular level, Western blot analysis was performed. Proteins were extracted from MDA-MB-231 cells treated with UD (4% *v*/*v*) and cisplatin (15 μM), individually or in combination, for 48 h. These specific concentrations were used based on the most pronounced effect of UD in promoting the pro-apoptotic effect of cisplatin revealed by flow cytometry. A significant increase in cleaved poly (ADP-ribose) polymerase (PARP) was observed in combination therapy compared to each treatment alone, with a 9-fold increase and a 1.5-fold increase upon combination and compared to UD and cisplatin alone, respectively (Figure 4C). 

To further elucidate the enhanced activation of the apoptotic pathway, the expression of pro-apoptotic protein Bax and the antiapoptotic protein Bcl-2 was evaluated, revealing an increase in the expression level of Bax, along with a decrease in Bcl-2 protein expression upon exposure to UD and cisplatin alone or in combination. Consequently, the ratio of Bax/Bcl-2 was determined, showing a significant increase by approximately twofold when comparing 15 μM cisplatin treatment to its combination with 4% *v/v* UD (Figure 4B). 

## 3. Discussion

Natural compounds have been reported to possess chemopreventive activities, highlighting their potential in cancer therapeutics [38]. *Urtica dioica* has been previously established to be used in folk medicine for the treatment of various ailments, and particularly in the treatment of cancer as an alternative and complementary approach [23]. The anticancer properties of nettle tea have previously been demonstrated on AML cells in vitro by our laboratory [27]. Considering that breast cancer is of the metastatic forms, associated with high incurability and mortality, it constitutes an interesting area of focus for cancer research and therapy [39]. Currently, scientists are focusing on the promising effect of combining natural products with chemotherapeutic drugs; hence, in this work, we evaluated the effect of UD in promoting the antitumor effects of such agents. While the anti-tumor potential of *Urtica dioica* has been studied before, many of these works used organic extracts of the plant. To our knowledge, this paper is the first to focus on the effect of an edible, aqueous UD extract in combination with cisplatin, a known chemotherapeutic drug, on the proliferation and growth of the most aggressive breast cancer cell line, the triple negative MDA-MB-231. Using an aqueous extract or a tea highlights its potential to be used in cancer prevention and treatment as it can easily be included in the daily diet, considering that tea is the second most popular beverage following water, widely consumed worldwide and commonly used in folk medicine due to its medicinal properties [15]. 

In our study, the aqueous UD extract used alone inhibited the proliferation of the MDA-MB-231 breast cancer cell line in a dose- and time-dependent manner, with the IC_50_ of 6.459% *v/v* for 24 h. This is in accordance with previous results, in which Mohammadi et al. demonstrated that a dichloromethane extract of UD inhibited the growth of MDA-MB-468 cells by inducing apoptosis, with no significant inhibition on normal L929 cells [40]. In another study, an aqueous extract of UD displayed a selective inhibitory effect on the proliferation of the U937 and KG-1 cell lines, with no major effect on the viability of the normal human B-lymphocyte cell line [27]. This clearly confirms the safety of aqueous UD infusions. We hereby present data that show that UD, when applied in combination with cisplatin, significantly enhanced the effect of the chemotherapeutic drug on the MDA-MB-231 cell line. Previously, D’Abrosca et al. demonstrated that UD enhanced the cytotoxicity of cisplatin in NSCLC cells through an endoplasmic reticulum-stress-mediated apoptosis [36] and enhanced the sensitivity of MDA-MB-468 cells to paclitaxel drug by inhibiting their growth and migration [35]. These studies highlight the potential of UD to be used in combination with chemotherapeutic drugs, as it enhances their therapeutic effect and reduces their adverse effects.

To assess the cell death mechanism responsible of the cytotoxic effect of UD in combination with cisplatin, cell death ELISA was performed to quantify DNA fragmentation which is a major hallmark of apoptosis. During programmed cell death, chromosomal DNA is cleaved into oligonucleosomal size fragments by endonucleases [31,41]. Our results showed a significant increase in DNA fragmentation when MDA-MB-231 cells were treated with the combination compared to each treatment alone. Similar results were previously reported on U937 cells, where an aqueous extract of UD caused an increase in the enrichment factor, and thus DNA fragmentation [27]. Moreover, the promising effect of UD was further confirmed on a molecular level, whereby we reported a significant upregulation in the expression of the cleaved form of the DNA repair enzyme, c-PARP. This is in accordance with the increase in PARP cleavage obtained in human prostate carcinoma LNCaP cells after 24 h of exposure to an aqueous UD extract [31]. 

Apoptosis is characterized by the flipping of phosphatidylserine from the inner leaflet of the cytoplasmic membrane to the outer one, allowing Annexin-V dye binding to the exposed phosphatidylserine. The results showed an increased percentage of apoptotic cells upon treating MDA-MB-231 cells with a combination of UD and cisplatin for 48 h compared to each of them separately. This result is in accordance with the increase in apoptotic U937 cells treated with UD as reported by Hodroj et al. [27]. To further confirm the activation of the apoptotic pathway at a molecular level, Western blot analysis of pro-apoptotic Bax and anti-apoptotic Bcl-2 proteins was performed [42]. In response to apoptotic stimuli, it appears that the Bax/Bcl-2 ratio holds more significance than the levels of either protein alone in the determination of apoptosis, which is why it has been described as a “rheostat” in cellular susceptibility to apoptosis [43,44,45]. More specifically, a predisposition to apoptotic stimuli has been shown to be indicated by an increase in the ratio of Bax/Bcl-2 within a cell, which is responsible for the subsequent cytosolic release of cytochrome c and the activation of downstream effectors of apoptosis [43]. In our study, the changes in the expression levels of Bax and Bcl-2 and the significant increase in the Bax/Bcl-2 ratio were indicative of the activation of the intrinsic pathway of apoptosis in the MDA-MB-231 cells treated with the combination of UD and cisplatin for 48 h compared to each treatment alone.

The chemical characterization of the aqueous UD extract has been previously performed by our lab as reported by Hodroj et al. Patuletin, m/p-hydroxybenzoic acid, and caffeic acid were the major compounds detected, which may be responsible for the anti-cancer and pro-apoptotic effects of UD, along with other components, such as bisabolol oxide B, gallic acid, chlorogenic acid, homovanillic acid, and kaempferol-3-O-rutinoside [27]. In fact, Zhu et al. demonstrated that patuletin induced apoptosis in the SK-BR-3 human breast cancer cell line through the inhibition of gene expression and activity of fatty acid synthase [46]. Reportedly, caffeic acid inhibited the proliferation of NSCLC H1299 cells and showed a synergistic anti-cancer effect when applied in combination with Paclitaxel [47], while its combination with Cisplatin exhibited synergism and inhibited the proliferation of human cervical cancer cell lines through the induction of apoptosis [48]. Therefore, we speculate that the promising results reported in our current study can be attributed to patuletin, m/p-hydroxybenzoic acid, and caffeic acid, previously characterized by LCMS/MS.

Overall, this study brings novelty to promising effects of UD tea infusion in promoting the effect of chemotherapeutic drugs. Our data suggest an enhanced sensitivity of the triple-negative MDA-MB-231 cell line to cisplatin treatment by a simple and edible leaf infusion of *Urtica dioica*, as reflected by an increase in apoptosis, DNA fragmentation, and changes in the expression levels of key apoptotic proteins. 

## 4. Methods

### 4.1. Urtica Dioica Aqueous Extract Preparation

As previously reported, *Urtica dioica* leaves were collected from Bawarij, Lebanon (33°49′0″ N 35°49′0″ E 1249 m above sea level) [27]. Briefly, the leaves were dried at room temperature, and the aqueous extract was prepared in type I autoclaved boiling water for 20 min. The nettle tea (5% *w*/*v*) was decanted in a beaker where the leaves were filtered using a cheesecloth. The obtained extract was then filtered using a syringe filter with a 0.45 µm pore size and further concentrated to reach a concentration of 10% of weight of leaves/volume (yield = 42 mg/mL) by Speed Vac, divided into 1 mL aliquots, and stored at −80 °C. On the day of treatment, the aliquots were thawed and applied to breast cancer cells using the appropriate concentrations. The chemical characterization of the UD aqueous extract has been previously performed by LCMS/MS and published by our laboratory [27]. A voucher specimen was deposited in the Beirut Arab University Herbarium (ID—RCED2019-363). 

### 4.2. Cell Culture

MDA-MB-231 breast cancer cells, obtained from ATCC, were cultured in Dulbecco’s Modified Eagle Medium (DMEM, Sigma-Aldrich, St. Louis, MO, USA) supplemented with 10% Fetal Bovine Serum (FBS, Gibco™, Dublin, Ireland) and 1% antibiotics (100 U/mL penicillin and 100 µg/mL streptomycin from Pen-Strep Lonza) [49]. The cells were observed daily under the ZOE fluorescent Cell Imager, split 3 times per week, and incubated at 37 °C and 5% CO_2_. The viability of the cells was also checked using the Trypan blue exclusion method.

### 4.3. MTS Cytotoxicity Assay

MDA-MB-231 cells were cultured in 96-well plates in triplicate at a concentration of 1 × 10^5^ cells/mL and incubated overnight. On the day of treatment, the cells were either treated with increasing concentrations of the UD aqueous extract (0, 1, 2, 3, 4, and 5% *v*/*v*), increasing concentrations of cisplatin (0, 5, 10, 15, 20 and 25 µM), or UD (4% *v*/*v*) in combination with a range of cisplatin (0, 5, 10, 15, 20 and 25 µM). Cells treated only with DMEM media served as control. Cell viability was assessed 24 and 48 h after treatment using the MTS reagent (Promega, Wiscosin, WI, USA). The conversion of tetrazolium salts to formazan by metabolically active cells was quantified by spectrophotometry (Varioskan™ LUX, Thermo Scientific, Waltham, MA, USA), where the absorbance of each well was measured at 492 nm as previously detailed by Khalil [50]. Cell viability was calculated by dividing the absorbance of the treated cells by the average absorbance of the untreated control cells. The half-maximal inhibitory concentration (IC_50_) was determined using the widely used non-linear regression curve of a dose-response variable slope via the Graphpad Prism version 9.1.0 [51,52,53,54].

### 4.4. Annexin V/Propidium Iodide Dual Staining

MDA-MB-231 cells were seeded in 6-well plates at a concentration of 0.7 × 10^5^ cells/mL overnight to reach a monolayer of approximately 80% confluency after 48 h. The cells were then treated with increasing concentrations of cisplatin (0, 5, 10, 15, 20, 25 µM) and in combination with 4% *v/v* UD (rationale for using 4% is detailed in the Results section). Untreated cells served as negative controls. After 48 h, the cells were detached using trypsin (Lonza), collected by centrifugation at 4 °C for 5 min, and the pellets were stained with Annexin V and Propidium Iodide (Annexin V-FITC Apoptosis Staining Kit, Abcam Inc., Cambridge, UK). The staining procedure was performed as previously described, according to the manufacturer’s instructions [55]. The samples were analyzed using the Guava easy Cyte™ flow cytometer. Upon the induction of apoptosis, phosphatidylserine flipped from the inner membrane leaflet to the outer leaflet. Living cells stained negative for both Annexin V and PI. Early apoptotic cells stained positive for only Annexin V, and late apoptotic cells stained positive for both. Total apoptotic cells were the sum of early and late apoptotic cells. 

### 4.5. Cell Death ELISA 

MDA-MB-231 cells were cultured in 6-well plates at a concentration of 0.7 × 10^5^ cells/mL and incubated overnight. The cells were treated with increasing concentrations of cisplatin (0, 15 and 20 µM) and in combination with 4% *v/v* UD. After 48 h, the cells were detached, lysed, and fragmented; cytosolic DNA was incubated in a previously prepared microplate coated with anti-histone antibodies of the Cell Death ELISA kit (Roche, Basel, Switzerland). Then, the wells were washed using the wash buffer provided, and the extracted DNA was incubated with anti-DNA antibodies tagged to a peroxidase enzyme. The wells were washed again, and the provided colorimetric substrate ABTS was added. The results were quantified by measuring the absorbance of each well after 15 min at 405 nm using the Varioskan™ LUX (Thermo Scientific, Waltham, MA, USA). This test is based on the idea that the presence of cytosolic nucleosomes, which are fragments of DNA wrapped around a histone core, can be used to quantify DNA fragmentation, which is a hallmark of apoptosis. Using anti-histone antibodies coated on the well of the plate and, subsequently, anti-DNA antibodies tagged with the enzyme, the Sandwich ELISA measured the DNA after collecting the cytosolic sections of the treated MBA-MB-231 cells. DNA fragmentation was quantified based on the calculation of the enrichment factor, which is the absorbance of the treated sample divided by the absorbance of the control untreated sample [56]. 

### 4.6. Western Blot 

MDA-MB-231 cells were plated in a 6-well plate at a concentration of 0.7 × 10^5^ cells/mL and incubated overnight. The cells in each well were treated differently: either with DMEM media only (control), with 15 µM cisplatin, with 4% *v/v* UD alone, or co-treated with 15 µM cisplatin and 4% *v/v* UD. After 48 h, cells were lysed using the RIPA buffer and protease inhibitor cocktail (MP Biochemical, California, USA). Proteins were collected, and quantification was performed using the Lowry method. Proteins were resolved by SDS-PAGE and the gels were blotted onto PVDF membranes. The membranes were blocked with 5% skimmed milk and then incubated with primary antibodies: anti-actin (Santa Cruz, Dallas, TX, USA), anti-Bax (Invitrogen, Waltham, MA, USA), anti-Bcl2 (Invitrogen, Waltham, MA, USA) and anti-cleaved Parp (Invitrogen, Waltham, MA, USA). After washing, the membranes were treated with the appropriate secondary antibodies for 1 h at room temperature (BioRad, Hercules, CA, USA). The membranes were washed again, and images were generated using the Clarity™ Western ECL Substrate on the ChemiDoc machine (BioRad, Hercules, CA, USA). To quantify blot bands and to calculate the relative expression of proteins, the ImageJ software was used [57].

### 4.7. Statistical Analysis

All experiments were performed in triplicate at three independent times. The calculations were performed in Excel, and the statistical significance was analyzed using the GraphPad Prism Version 9.1.0 by performing one-way or two-way ANOVA depending on the experiment. The results were reported as mean +/− SD (the standard error of the mean). The statistical difference was set at * *p* < 0.5, ** *p* < 0.05, *** *p* < 0.001.

## 5. Conclusions and Future Perspectives

This study lays the foundation for future work on cancer treatment as it is the first to investigate the effect of UD in combination with cisplatin on the most aggressive breast cancer cell line. The aqueous extract of *Urtica dioica* enhanced the sensitivity of the triple negative MDA-MB-231 breast cancer cell line to cisplatin in vitro. This was reflected by the enhanced decrease in proliferation due to the induction of apoptosis, which was accompanied by an increase in DNA fragmentation and upregulation of c-PARP, along with an increase in the Bax/Bcl-2 ratio, indicating the activation of apoptosis. We can observe a promising effect of UD in combination with cisplatin, highlighting its potential to be used in alternative medicine along with chemotherapeutic drugs to enhance the sensitivity of cancer cells to the drugs and reduce the various side effects they cause. This is especially important considering that an aqueous extract was the focus of this paper, which emphasizes its ease of consumption and incorporation into the human diet as a simple infusion. Despite its promising potential, further studies are needed to confirm this effect on other types of aggressive cancers, in vivo and in clinical trials. Furthermore, studying the effect of UD major compounds in combination with cisplatin is crucial to further elucidate the bioactive molecules responsible for enhancing the sensitivity of breast cancer cells. Along with that, it is of a great interest to assess the effect of this combination treatment on cell invasion and metastasis, which would constitute another area of focus for future studies.

## Data Availability

All data generated and analyzed in this study are mentioned in this manuscript.

## Supplementary Materials

The following supporting information can be downloaded at: https://www.mdpi.com/article/10.3390/ph16060780/s1, Table S1: Cell viability of MDA-MB-231 in response to treatment with IC50 concentrations of UD and Cisplatin for 24 and 48 h.

## Abbreviations

AML	Acute Myeloid Leukemia
DMEM	Dulbecco’s Modified Eagle Medium
ELISA	Enzyme Linked Immunostaining Assay
FBS	Fetal Bovine Serum
HER2	Human Epidermal Growth Factors Receptor 2
IC50	Inhibitory Concentration
LC-MS	Liquid Chromatography/Mass Spectrometry
PARP	Poly-ADP ribose polymerase
PBS	Phosphate Buffered Saline
PI	Propidium Iodide
PVDF	Polyvinylidene fluoride
RIPA	Radio-Immunoprecipitation assay
