# Supplementary material for: Urtica dioica Leaf Infusion Enhances the Sensitivity of Triple-Negative Breast Cancer Cells to Cisplatin Treatment
Source: Pharmaceuticals (Basel). 2023 May 23;16(6):780. doi: 10.3390/ph16060780 (PMC10302233; doi:10.3390/ph16060780)
Supplement: Supplementary file 1 [file pharmaceuticals-16-00780-s001.zip › pharmaceuticals-2380964-supplementary.pdf]

Urtica dioica leaf infusion enhances the sensitivity of triple-negative breast cancer cells to cisplatin treatment

Guy Nafeh[1], Maria Abi Akl[1†], Jad Samarani[1†], Rawane Bahous[1], Georges Al Kari[1], Maria Younes[1], Rita Sarkis[1,2] and Sandra Rizk[1*]

*Correspondence: Corresponding author: sandra.rizk@lau.edu.lb

Supplementary Table (S1)

	UD extract		Cisplatin	
**IC_{50} concentration**	6.46 %v/v (24hours)	4.16%v/v (48hours)	32.21uM (24hours)	12.76uM (48hours)
**Percent Viability**	51.37 ± 2.55	51.28 ± 5.08	51.06 ± 4.83	47.96 ± 6.37

Table S1*: Cell viability of MDA-MB-231 in response to treatment with IC_{50} concentrations of UD and Cisplatin for 24 and 48hours.*
